# Screening, Optimization, and Bioavailability Research of Natural Deep Eutectic Solvent Extracts from *Radix Pueraria*

**DOI:** 10.3390/molecules26030729

**Published:** 2021-01-31

**Authors:** Yan Huang, Jiehong Yang, Yu Zhao, Li Yu, Yu He, Haitong Wan, Chang Li

**Affiliations:** 1 College of Life Science, Zhejiang Chinese Medical University, Hangzhou 310057, China; 15119306088@163.com (Y.H.); zhaoyu9701@126.com (Y.Z.); yuli9119@126.com (L.Y.); 2 College of Basic Medical Sciences, Zhejiang Chinese Medical University, Hangzhou 310057, China; yjhong@zcmu.edu.cn; 3 College of Pharmacy, Zhejiang Chinese Medical University, Hangzhou 310057, China; heyu0923@sina.com

**Keywords:** natural eutectic solvent, *Radix Pueraria*, pharmacokinetic, oral bioavailability, HPLC-MS, green solvent, puerarin, extraction

## Abstract

Natural deep eutectic solvent (NaDES) is generally considered as a greener alternative to fossil solvent, with great potential in various areas. In the present work, 25 different NaDESs were screened for the extraction of puerarin (PUE) and its two natural derivatives from *Radix Pueraria* (RP). As the main isoflavone in RP, PUE has a wide range of biological activities. However, its application is restricted due to its poor solubility in water and low oral bioavailability. In this study, the extraction of PUE with NaDESs showed significant advantages compared with traditional solvents. While using L-Pro-Maa (L-proline-malic acid) under optimal conditions, the optimized yields of PUE, 3-MPR and PRX were 98.7 mg/g, 16.3 mg/g and 9.9 mg/g, respectively, which were 2.2-, 2.9- and 3.4-fold higher than that of water. Furthermore, the oral bioavailability of PUE in NaDES extracts was comparatively investigated in rats with HPLC-MS technique. Pharmacokinetic analysis revealed that the relative bioavailability of PUE in L-Pro-Maa extract is 323%. The result indicated that NaDES is not only a sustainable ionic liquid with higher extraction efficiency, but also an enhancer of oral bioavailability of specific natural products.

## 1. Introduction

The development of novel green solvents is one of the hot spots in green and sustainable chemistry [1]. As a particular type of ionic liquids (IL) firstly reported by Choi et al. in 2011 [2], natural eutectic solvent (NaDES) is essentially a eutectic solvent formed from a pair of naturally occurring hydrogen bond acceptors (HBAs) and hydrogen bond donor (HBDs). Compared with traditional fossil solvents and ordinary ILs, NaDESs possess the advantages of a greener resource [3], lower cost and toxicity [4], better biocompatibility, and bio-degradability [5]. Therefore, NaDESs have been recognized as a novel class of sustainable solvents and widely applied in analytical [6] and biomedical [7] research. Particularly, NaDESs have been intensively used in the extraction of various natural bioactive compounds to produce functional herbal products [8,9,10]. Moreover, studies have revealed that NaDES can not only perform as a better extraction solvent, but also an enhancer of other important functions. For instance, Wang et al. developed a highly efficient enzymatic hydrolysis reaction to convert rutin to isoquercitrin and L-rhamnose [11]. In our study, the oral bioavailabilities of hydroxysafflor yellow A and anhydrosafflor yellow B were greatly improved in the NaDES media [12].

**Figure 1:**
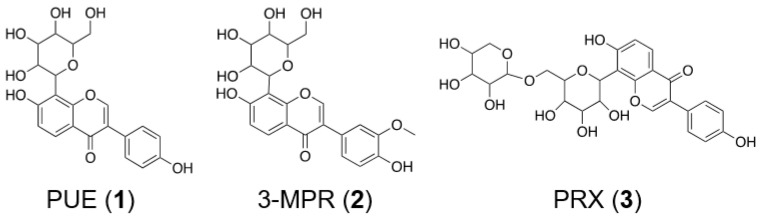
Chemical structures of puerarin flavones in this study.

*Radix Pueraria* (RP, known as *Gegen* in China) is the dried root of the leguminous plant *Pueraria lobata* (Willd.) Ohwi [13]. As one of the most widely used traditional Chinese medical plants and functional foods, RP has exhibited good potential in the treatment of pain [14], diabetes [15] and strokes [16]. Analytical chemistry research has revealed that the major ingredients in RP are isoflavones derivatives (Figure 1), including puerarin (PUE, 8-β-D-glucopyranose-4′,7-dihydroxyisoflavone), 3′-methoxypuerarin (3-MPR) and puerarin-6′′-O-xyloside (PRX). PUE was reported to have diverse pharmacological activities, including antidiabetic [17], cardiac fibrosis inhibitive [18], and hepatoprotective [19] effects. In addition, although pharmacological research on PRX and 3-MPR is relatively rare, PRX showed great potential in suppressing tumor growth as a prodrug [20], and 3-MPR exhibited protective effects on cerebral ischemia-reperfusion injuries [21].

Despite the various biological benefits of the major constituents from PR, the bioavailabilities of PUE, 3-MPR and PRX are relatively poor. The oral bioavailability of PUE was determined as about 7% [22]. Efforts have been devoted into the improvement of the solubility and bioavailability of *Pueraria* flavones. Zhang et al. [23] developed an oral drug nanocrystal self-stabilized Pickering emulsion (NSSPE) and investigated the oral bioavailability of PUE NSSPE. The results showed that the C_max_ of PUE was increased from 634.17 ng/mL to 3226.14 ng/mL. Qiao et al. [24] developed self-microemulsifying drug-delivery systems (SMEDDS). The C_max_ of PUE after oral administration reached up to 1.67 μg/mL, comparing with 1.03 μg/mL when PUE aqueous suspension was administrated. Herein, inspired by the previous reports of the potential of NaDES in enhancing extraction efficiency and oral bioavailability, we systematically investigated optimization of the screening and extraction parameters and pharmacokinetic behaviors of *Pueraria* flavones in RP with the help of NaDESs.

## 2. Results

### 2.1. Screening of NaDESs for the Extraction of PUE, 3-MPR and PRX

**Figure 2:**
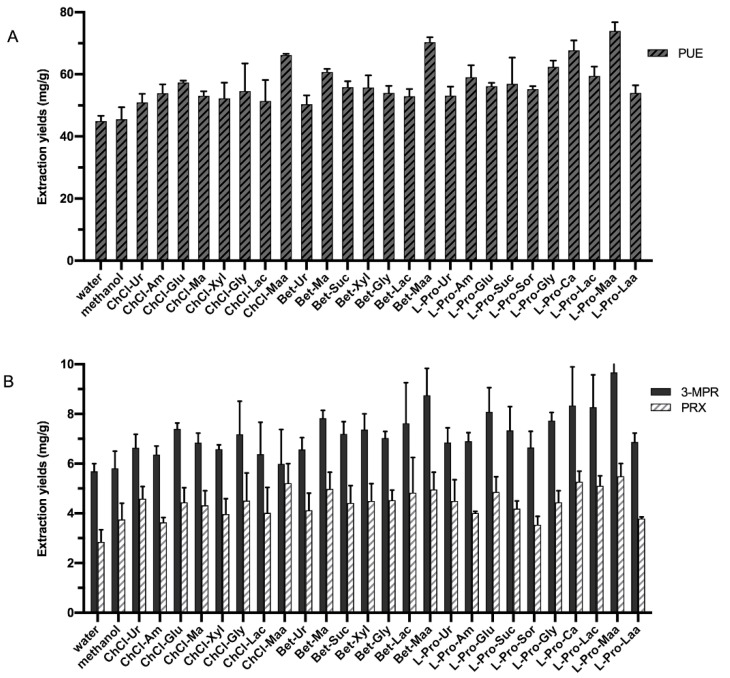
Extraction yields of puerarin (PUE), 3′-methoxypuerarin (3-MPR), and puerarin-6′′-O-xyloside (PRX) from *Radix Pueraria* (RP) using water, methanol, and 25 different natural deep eutectic solvents (NaDESs). (**A**) Extraction yields of PUE in different solvents; (**B**) Extraction yields of 3-MPR and PRX in different solvents.

In order to screen for NaDESs with high efficiency in extraction of *Pueraria* flavones, three routine HBAs were used, including choline chloride, betaine, and L-proline. The representative HBDs were carbohydrates (glucose, maltose, sucrose), alcohols (xylitol, glycerol), organic acids (lactic acid, malic acid) and organic bases (urea, acetamide). In total, 25 different NaDESs were prepared and their extraction effects on PUE, 3-MPR and PRX were determined together with water and methanol for comparison (Figure 2 and Table S2 in the Supplementary Materials). In the initial screening, the extraction factors were set as follows, solid/liquid (S/L) ratio 25 mg/mL, NaDES content 75%, extraction temperature 50 °C, and the extraction time of 30 min. As depicted in Figure 2A & B, water and methanol exhibited almost the same effect in extraction of PUE (44.9 and 45.5 mg/g) and 3-MPR (5.6 and 5.8 mg/g), while the yield of PRX was slightly higher in methanol (2.8 and 3.8 mg/g). To our delight, most NaDESs contributed to higher yields of PUE, 3-MPR and PRX compared with methanol and water. By comparing among the three HBAs, it was found that L-Pro-based NaDESs showed slightly higher efficiency than that of ChCl- and Bet-based NaDESs. On the other hand, the type of HBD exhibited a greater impact on the extraction capacities of NaDESs. Maa-based NaDESs, including L-Pro-Maa, Bet-Maa and ChCl-Maa, showed better extraction abilities. Among them, L-Pro-Maa was selected as the best extraction solvent, with the extraction yield of PUE at 74.0 mg/g, 3-MPR at 9.7 mg/g, and PRX at 5.5 mg/g. In addition, the calibration curves, linear ranges, LOD and LOQ for the analytes by HPLC are all listed in Table S4 in the Supplementary Materials.

### 2.2. Optimization of the Extraction Factors

**Figure 3:**
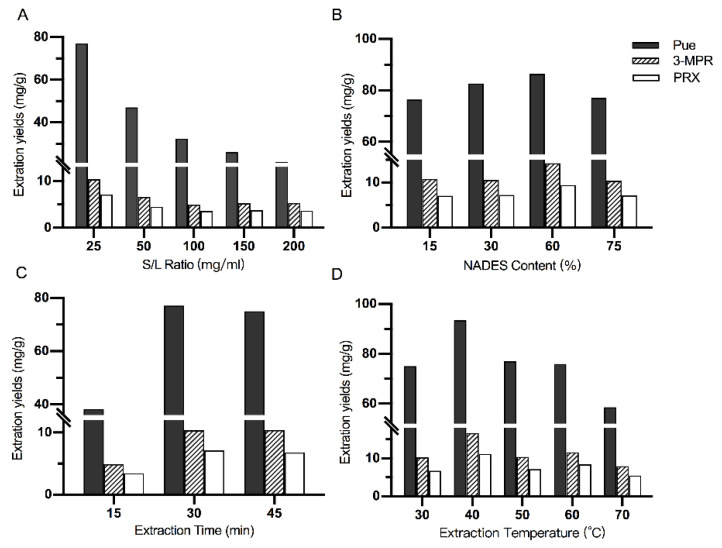
Extraction yields of PUE, 3-MPR and PRX from RP using L-Pro-Maa with different parameters: (**A**) solid/liquid (S/L) ratio; (**B**) NaDES content; (**C**) Extraction time; (**D**) Extraction temperature.

The efficiency of ultrasonic-assisted extraction (UAE) processes on herbal materials is usually affected by a lot of factors; therefore, four key extraction parameters were optimized using a single factor experiment design with L-Pro-Maa as the extraction solvent. The default extraction parameters were set as: S/L ratio 25 mg/g, extraction temperature 50 °C, extraction time 30 min, and NaDES content 75%. As summarized in Figure 3, the effects of S/L ratio, NaDES content (%), extraction temperature, and extraction time were evaluated. In the optimization of S/L ratio ranging from 25, 50, 100, 150 and 200 mg/mL, 25 mg/mL was observed to have the highest extraction yields of all of the three flavones (Figure 3A). For NaDES content, 15%, 30%, 60% and 75% NaDES (*v*/*v*) were testified (Figure 3B). It was observed that increasing NaDES content would lead to higher yields of PUE, 3-MPR and PRX, while the maximum extraction efficiency was obtained when the NaDES content reached 60%. This may be due to the increasing viscosity of the solvent when the content of NaDES increases, which hindered the dispersion of PR particle and further the extraction yields of the flavones [6]. Extraction time and temperature are also important factors in most UAE processes. The results indicated that increased extract time improved the extraction yield until it reached 30 min, which was selected as the optimal extraction time (Figure 3C). The effect of extraction temperature was in a similar manner, where 40 °C was considered as the most applicable parameter (Figure 3D). Finally, the optimal extraction factors were as follows: S/L ratio at 25 mg/g, extraction temperature at 40 °C, extraction time at 30 min, and NaDES content at 60%.

### 2.3. HPLC-MS/MS Method Validation

Accumulating research has indicated that NaDESs have broader functions other than the improvement of the extraction efficiency. Inspired by the previous work that NaDES enhanced the bioavailability of berberine [25], hydroxysafflower yellow A and anhydrosafflor yellow B [12], we next investigated the in vivo pharmacokinetics of NaDES and aqueous extracts of PR comparatively. The content of PUE is more than 10-fold higher than that of 3-MPR and PRX; therefore, only the plasma concentrations of PUE were measured. At the beginning, an HPLC-MS method was established and validated using a Q-Exactive mass spectrometry system. Selected ion monitoring (SIM) mode was selected herein, in consideration that the other puerarin flavones might produce fragments with the same *m/z* as PUE and provide false positive signals in target MS^2^ mode. In addition, the high resolution of Q-Exactive could provide enough sensitivity and precision in the determination of PUE.

#### 2.3.1. Specificity

**Figure 4:**
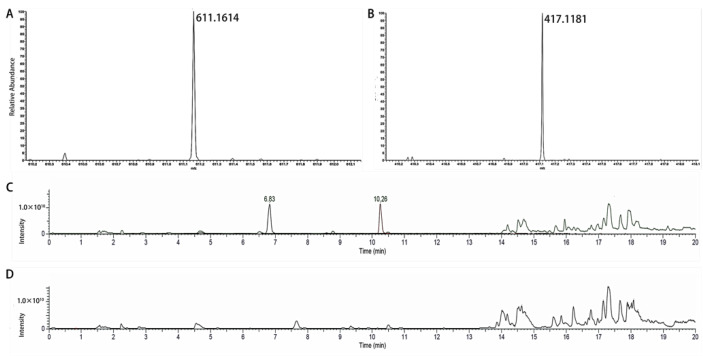
Mass spectra of IS (**A**) and PUE (**B**); Total ion chromatogram of plasma spiked with PUE and IS (**C**); Total ion chromatogram of blank plasma (**D**).

The representative chromatograms of blank plasma and blank plasma spiked with analytes and internal standard (IS) are displayed in Figure 4. The method was observed to be highly selective for PUE in plasma, with the retention times of PUE and IS at 6.78 and 10.26 min, respectively. Under the established chromatographic condition, there was no endogenous interference in the plasma.

#### 2.3.2. Linearity

The calibration curve was established by plotting the peak area ratio of PUE to IS (*y*) versus the rat plasma PUE concentration (*x*). The regression equation was *y* = 2.4262 × *x* + 0.0295 with a correlation coefficient (*r*^2^) > 0.99. The calibration curve was linear in peak area ratios over the concentration range from 0.01 mg/L to 10 mg/L.

#### 2.3.3. Precision and Accuracy

The intra- and inter-day precision and accuracy were determined by analyzing the quality control (QC) samples at the concentration of 0.1, 1 and 10 mg/L. All values are listed in Table 1. The assay values were all within the acceptable range, and the method was considered accurate and precise.

#### 2.3.4. Recovery and Matrix Effect

The matrix effect and the recovery of the PUE were evaluated and are listed in Table 2. All values of the recovery were between 91.9% and 106.9%. The matrix effects were between 103.2% and 107.3%. The results clearly demonstrated that the extraction recoveries of samples were stable and found to be within the acceptable range, and the plasma matrix effect was negligible in the assay. To avoid carryover, an injection of methanol before every sample run was performed, and no interfering or re-appearing peak was observed under the analytical conditions.

### 2.4. Pharmacokinetic Analysis

**Figure 5:**
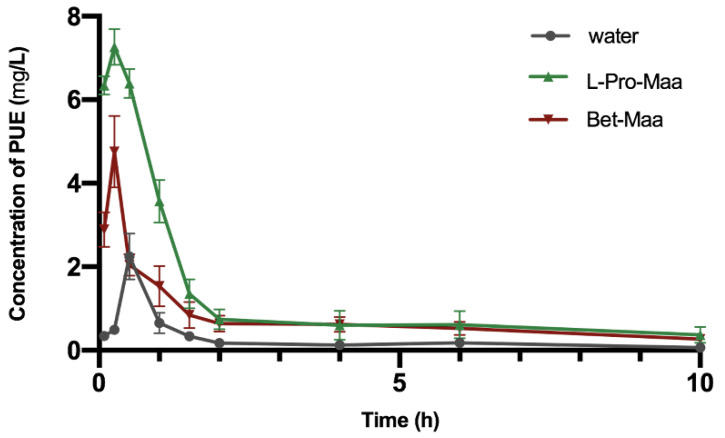
Time course of plasma concentrations (mean ± SD) of PUE in Sprague-Dawley rats following oral administration of 2 g/kg RP in L-Pro-Maa(green), Bet-Maa (red) and water (grey). The error bars represent the standard error base on the plasma concentrations three rats in same group.

Two kinds of NaDES with high extraction efficiency of the three puerarin flavones, namely, L-Pro-Maa and Bet-Maa, were selected to perform in vivo pharmacokinetic research together with water. The three extracts were orally administered to Sprague-Dawley rats at a dose of 2.04 g RP/kg. The plasma levels of PUE up to 10 h after administration were determined with the HPLC-MS method and then used to calculate the pharmacokinetic parameters. The time course of plasma concentrations of PUE was plotted in Figure 5, and the main pharmacokinetic parameters are listed in Table 3.

In all of the solvents (NaDESs and water), the plasma concentration–time curve of PUE showed a single peak after the RP extract was administered to rats. It was observed that PUE was quickly absorbed and eliminated in 10 h. The concentration of PUE reached maximum within 0.5 h. The T_max_ values of the three experiments revealed that there are delays of time when the concentration of PUE reached maximum. The T_1/2z_ is an indicator of the elimination rate of drugs. The results showed that L-Pro-Maa helped to extend the lifespan of PUE in vivo. The other important pharmacokinetic parameters, C_max_, AUC_(0–t)_ and AUC_(0–∞)_, could directly reflect the bioavailability of drug and the effect of pharmaceutics. It is shown that Bet-Maa extract showed a remarkable increase in the values of C_max_, AUC_(0–t)_ and AUC_(0–∞)_ compared with that of aqueous extract. The value of F_r_ in Bet-Maa group was calculated as 212%. L-Pro-Maa exhibited an even higher bioavailability, because the values of C_max_, AUC_(0–t)_ and AUC_(0–∞)_ were further increased. The C_max_ reached up to 7.267 ± 0.432 mg/L, which is more than three times higher than that of water group. The relative bioavailability was calculated as 323%, which is higher than that of the previously reported NSSPE [23] and SMEDDS [24] methods.

## 3. Discussion

NaDES has been proved as an efficient extraction media for natural products with low toxicity and environmental friendliness. In the present study, the yield of PUE extracted by L-Pro-Maa from RP under optimal conditions was 98.7 mg/g, and the extraction rates of 3-MPR and PRX were 16.3 mg/g and 9.9 mg/g, respectively, which was 2.2-, 2.9- and 3.4-fold higher than that of water. This might be more strong evidence to prove the capacity of NaDES for its application in the nutraceutical and pharmaceutical industries. In the screening for NaDES, it was found that while acidic compounds were used as HBD, the corresponding NaDESs usually showed better extraction effects toward PUE derivatives. This phenomenon could be attributed to the intense hydrogen bond connection between organic acids and the phenolic target compounds.

Particularly, two NaDESs, namely L-Pro-Maa and Bet-Maa, which showed high efficiency for the extraction of the three PUE derivatives, were employed to evaluate the effect of NaDES for the oral bioavailability of PUE. It is noteworthy that the rats administrated with NaDES extracts were observed in normal situations without any symptoms of toxicosis. Together with our work on chalcone derivatives from *Carthamus tinctoriu* [12] and a previous report on berberine [25], the present study might provide a new beneficial property of NaDES, and the capacity to enhance the oral bioavailability of compounds. Especially, this might be useful for naturally occurring nutrients such as resveratrol and curcumin, the clinical applications of which were greatly hindered by their poor bioavailabilities. However, the mechanism of the enhancement is still unclear and is worth investigating in future.

In conclusion, a green and efficient extraction method was developed, and the PUE in rat plasma was determined by HPLC-MS to compare the bioavailability of the active ingredients extracted by different extraction method. L-Pro-Maa was selected as the best solvent. With the help of the HPLC-MS technique, the pharmacokinetic parameters of PUE after oral administration to rats were obtained. Further calculation revealed that the low oral bioavailability of PUE was significantly improved in NaDES extract of PR, with the relative bioavailability reaching up to 323%. Thus, NaDESs as a novel extraction method used to extract traditional Chinese medicine were not only greener and more efficient, but also a way to enhance the absorption and oral bioavailability of natural active ingredients.

## 4. Materials and Methods

### 4.1. Chemicals

Dried RP samples were purchased from a local traditional Chinese medicine market (Hangzhou, China). PUE (>98%) and rutin (>98%) were purchased from Shanghai Yien Chemical Technology Co., Ltd. (Shanghai, China). The compounds 3-MPR (>98%) and PRX (>98%) were purchased from Chengdu Alfa Biotech Co., Ltd. Compounds for NaDES preparation and formic acid (>88%) were all purchased from Shanghai Aladdin Bio-Chem Technology Co., Ltd. (Shanghai, China). HPLC-grade methanol was purchased from Tedia Co. (Fairfield, OH, USA), and the deionized water used in the study was obtained from a Milli-Q water purification system (Bedford, MA, USA).

### 4.2. Animals

Male Sprague-Dawley rats weighing 280–300 g were obtained from the Experimental Animal Center of the Zhejiang Chinese Medical University, Hangzhou, China (Laboratory animal certificate: SYXK (Zhe) 2018-0012). All protocols in this study were approved by the Animal Subjects Review Board of Zhejiang Chinese Medical University (No. 20180514-04, 14 May 2018). The Guidelines for the Care and Use of Laboratory Animals, prepared by the National Academy of Sciences (NIH publication No. 85-23, revised 1996) were followed to care for all animals. The present research complied with the commonly accepted “3Rs”. The rats were bred in a breeding room at 24 ± 2 °C with 60 ± 5% humidity and a 12 h dark–light cycle with free access to food and water. All of experimental rats were fasted overnight before the experiments.

### 4.3. Preparation of RP Extracts

All of the NaDESs were prepared following the previously reported method [26], with modifications. The compositions of NaDESs are listed in Table S1. In brief, HBA, HBD, and water were mixed in corresponding ratios. The mixtures were then stirred at 90 °C until a homogeneous liquid was formed. All NaDESs were kept in a desiccator before usage.

For the RP extract, the dried herb samples were pulverized to uniform size. Then, 25 mg of the powder was accurately weighed in a 1.5 mL centrifuge tube and extracted with 1 mL of corresponding solvent (water, methanol or NaDES–water mixed solvent). The UAE process was carried out with an ultrasonic bath (KQ5200DE, Kunshan Ultrasonic Instrument Co, Suzhou, China) at the frequency of 50 kHz. After centrifugation for 16 min at 13,300 rpm, the clear supernatant was collected and diluted three times with methanol–water (1:1) prior to HPLC analysis. For optimization of the extraction procedure, corresponding factors were changed, respectively.

### 4.4. HPLC Analysis of RP Extracts

The HPLC analysis was performed on an Agilent 1200 system equipped with a G1311A QuatPump (Agilent Technologies, Inc., Santa Clara, CA, USA), a G1322A degasser, a G1315D diode array detector (DAD), and a G1329A ALS with a 20 μL loop. An Hypersil ODS-C18 (250 mm × 4.6 mm i.d., 5 μm, Agilent Technologies, Inc., Santa Clara, CA, USA) column was used. The mobile phase (phase A) was 0.1% formic acid solution (*v/v*)—phase B was methanol. A gradient program was used as follows: 0–10 min, 20–30% B; 10–15 min, 30% B; 15–35 min, 30–95% B; 35–45 min, 95% B. The flow rate was 0.8 mL/min, and the injection volume was 10 μL. The chromatograms were recorded at 254 nm.

In addition, stock standard solutions of PUE, 3-MPR and PRX were prepared by dissolving in the analytical grade methanol for quantification.

### 4.5. Establishment of HPLC-MS Method for Plasma Analysis

HPLC-MS analysis was performed on an Ultimate 3000 HPLC system (Thermo Fisher Scientific Co., Ltd., Waltham, MA, USA) coupled to a Q-Exactive hybrid quadrupole-orbitrap mass spectrometer (Thermo Fisher Scientific Co., Ltd., Waltham, MA, USA). The chromatographic separation was achieved using a Poroshell 120 EC-C18 column (3.0 mm × 150 mm, 2.7 μm, Agilent Technologies, Inc., Santa Clara, CA, USA). The mobile phase (phase A) was 0.1% formic acid solution (*v/v*)—phase B was acetonitrile. A gradient program was used as follows: 0–10 min, 10–25% B; 10–15 min, 25–95 % B. The flow rate was 0.4 mL/min, and the injection volume was 20 μL.

For the measurement of PUE, the mass spectrometer operated in ESI (electrospray ionization)-positive mode using single ion monitoring (SIM). The *m/z* data—611.1614 (IS) and 417.1181 (PUE)—were recorded. The parameters of the mass spectrometer were set as follows: capillary temperature, 256 °C; S-lens RF level, 55.0; spray voltage, 3.8 kV; automatic gain control (AGC) target, 5 × 10^4^; sheath gas flow rate, 45; aux gas flow rate, 11; aux gas heater temperature, 400 °C. The isolation window was set at 2.0 Da and the resolution was set 70,000. Xcalibur software (v4.1, Thermo Fisher Scientific Co., Ltd., Waltham, MA, USA) was used for quantitative data processing. The quantification analysis was conducted by the area ratio of each species to that of the internal standard.

### 4.6. Validation of HPLC-MS Method for Plasma Analysis

The method was validated by determining its specificity, linearity, recovery, accuracy, precision, and matrix effect.

Linearity was determined by plotting the peak-area ratio of PUE to IS versus PUE concentration. The linearity of the standard curves was calculated by linear regression (*y* = a × *x* + b). A coefficient of determination (*r*^2^) more than 0.99 was considered acceptable for quantification.

Accuracy and precision were assessed by measuring QC samples at three concentration levels. The intra-day precision was demonstrated by analyzing six replicates at each concentration level in one day, and inter-day precision by analyzing three replicates of each concentration over three consecutive validation days. The precision was expressed as RSD and the accuracy as RE. RSD and RE should not have exceeded ±15%.

The recovery was evaluated on the PUE of three concentration of QC samples and the IS at the concentration of 1 mg/L, and each sample was repeated three times. The matrix effect was investigated by comparing the PUE peak area results of samples spiked with PUE standard solutions to those of the pure reference standard solutions.

The specificity was evaluated by comparing the chromatograms of blank plasma samples obtained from rats with plasma spiked with analytes and IS, as well as plasma samples after an oral dose. All blank plasma samples were prepared, which ensured the absence of interfering peaks.

### 4.7. Pharmacokinetic Study

The Sprague-Dawley rats were randomly divided into three groups (*n* = 3). Group 1 received the RP extract extracted by water, group 2 received the RP extract extracted by Bet-Maa, and group 3 received the RP extract extracted by L-Pro-Maa. All rats were orally administrated with RP extracts at a dose of 2.04 g/kg [27]. Blood samples (300 μL) taken from the orbital vein at 0.083 (5 min), 0.25, 0.50, 1, 1.5, 2, 4, 6 and 10 h after administration were collected in heparinized tubes, which were centrifuged at 4000 rpm for 10 min at 4 °C. The supernatant plasma was transferred into clean tubes and kept at −20°C.

Before HPLC-MS analysis, the samples were thawed under room temperature. A volume of 100 μL rat plasma was spiked with 10 μL of IS solution (equivalent to 1 μg/mL for 100 μL plasma sample) followed by the addition of 300 μL methanol to precipitate protein. The mixture was vortex-mixed for 30 s, and centrifuged at 12,000 rpm for 12 min at 4 °C. The supernatant was transferred into another clean tube, dried under nitrogen, and reconstituted with 100 μL methanol.

Pharmacokinetic parameters for PUE were calculated based on the plasma concentration versus time data using Drug and Statistic Version 3.2.6 (DAS 3.2.6) software (the Mathematical Pharmacology Committee, Chinese Pharmacological Society, Shanghai, China). The peak time (t_max_) and maximum plasma concentration of PUE (c_max_) were determined directly from the mean plasma concentration-time curve (C–T curve). T_1/2z_ was calculated by the non-compartmental model as t_1/2z_ = 0.693/zate, where zate is the slope of the terminal phase of the C–T curve calculated by linear regression. The area under the C–T curve (AUC_0–t_) and the area under the C–T curve from zero to infinity (AUC_(0–∞)_) were calculated using the linear trapezoidal rule. All of the values are expressed as the means ± standard deviations (SD). Mean residence time (MRT) was calculated as AUC/AUMC, where AUMC is the area under the first moment curve. The relative bioavailability (F_r_) was calculated with the aqueous extract of RP as a control. Relative bioavailability of NaDES extracts versus aqueous extract was estimated by the ratio of AUC after oral administration of NaDES extract to AUC after oral administration of the aqueous extract.

## Figures and Tables

**Table 1:** Intra- and inter-day precision and accuracy at different concentrations. (*n* = 6).

Analytes	Concentration (mg/L)	Measured (mg/L)	Intra-Day		Inter-Day	
			RSD (%)	RE (%)	RSD (%)	RE (%)
PUE	0.1	0.1 ± 0.0	9.9	13.2	8.0	5.3
	1	0.9 ± 0.1	7.8	−11.2	14.1	−1.0
	10	10.0 ± 1.3	12.7	5.5	13.9	9.8

RSD: precision; RE: accuracy.

**Table 2:** The recoveries and matrix effects of PUE in rat plasma.

Analytes	Nominal Concentration (mg/L)	Matrix Effect (%)	Recovery (%)
PUE	0.1	103.2 ± 3.7	106.9 ± 7.4
	1	106.3 ± 4.8	91.9 ± 4.6
	10	107.3 ± 7.7	97.4 ± 9.3

**Table 3:** Main pharmacokinetics parameters of PUE after oral administration of the RP extracts with water, Bet-Maa and L-Pro-Maa.

Parameters	PUE		
	Water	Bet-Maa	L-Pro-Maa
T_max_ (h)	0.5	0.25	0.25
C_max_ (mg/L)	2.248 ± 0.552	4.757 ± 0.854 *	7.267 ± 0.432 *
T_1/2z_ (h)	6.146 ± 1.621	5.143 ± 0.817	7.281 ± 3.934
AUC_(0–t)_ (mg/L × h)	2.623 ± 0.642	7.453 ± 1.267 *	11.869 ± 2.181 *
AUC _(0–∞)_ (mg/L × h)	3.243 ± 0.742	9.483 ± 1.897	16.48 ± 5.714 *
MRT_(0–t)_ (h)	2.681 ± 0.047	3.122 ± 0.163	2.405 ± 0.689
F_r_ (%)	100	212	323

*: *p* < 0.05 compared with water group.

## Data Availability

The data that support the findings of this study are available from the corresponding author, upon reasonable request.

## Supplementary Materials

The following are available online: Table S1: Different ratios of composition of NaDESs; Table S2: Extraction yields of PUE, 3-MPR and PRX from RP using NaDESs and traditional solvents; Table S3: Extraction yields of PUE, 3-MPR and PRX from RP using L-Pro-Maa under different conditions; Table S4: Calibration curves, linear ranges, LOD and LOQ for analytes by HPLC; Figure S1. HPLC chromatogram of aqueous extract of RP; Figure S2: HPLC chromatogram of L-Pro-Maa extract of RP; Figure S3: HPLC chromatogram of methanol extract of RP.
