# Supplementary material for: Screening, Optimization, and Bioavailability Research of Natural Deep Eutectic Solvent Extracts from Radix Pueraria
Source: Molecules. 2021 Jan 31;26(3):729. doi: 10.3390/molecules26030729 (PMC7866862; doi:10.3390/molecules26030729)

Screening, Optimization and Bioavailability of Natural Deep Eutectic Solvent Extracts from *Radix Pueraria*

Yan Huang[#,1], *Jiehong Yang*[#,1], *Yu Zhao* [1], *Li Yu* [1], *Yu He* [1], *Haitong Wan**[,1], *Chang Li**[,1]

[1] Zhejiang Chinese Medical University, Hangzhou, 310057, PR China

[#] These authors contributed equally to this work.

[*] Corresponding Authors

[*] Chang Li, lichang@zju.edu.cn; Tel./Fax: +86 571 86613716

[*] Haitong Wan, whtong@126.com; Tel./Fax: +86 571 86613716

Supporting Information

Table of contents

Table of contents	Page
Table S1. Different ratios of composition of NaDESs	3
Table S2. Extraction yields of PUE, 3-MPR and PRX from RP using NaDESs and traditional solvents	4
Table S3. Extraction yields of PUE, 3-MPR and PRX from RP using L-Pro-Maa under different conditions	5
Table S4. Calibration curves and linear ranges for analytes by HPLC	6
Figure S1. HPLC chromatogram of water extract of RP	7
Figure S2. HPLC chromatogram of L-Pro-Maa extract of RP	7
Figure S3. HPLC chromatogram of methanol extract of RP	8

Table S1. Different ratios of composition of NaDESs

No.	NaDES	Type of HBA	Type of HBD	HBA/HBD ratio(water)
1	ChCl-Ur	Choline Chloride	Urea	1:2
2	ChCl-Am		Acetamide	1:1:(1)
3	ChCl-Glu		D-Glucose	1:1:(2)
4	ChCl-Ma		Maltose	2:1:(4)
5	ChCl-Xyl		Xylitol	5:2:(5)
6	ChCl-Gly		Glycerol	1:2
7	ChCl-Lac		Lactic Acid	1:1
8	ChCl-Maa		DL-Malic Acid	1:1
9	Bet-Ur	Betaine	Urea	1:1:(2)
10	Bet-Ma		Maltose	5:2:(6)
11	Bet-Suc		Sucrose	2:1:(7)
12	Bet-Xyl		Xylitol	1:1:(1)
13	Bet-Gly		Glycerol	1:1
14	Bet-Lac		Lactic Acid	1:1:(1)
15	Bet-Maa		DL-Malic Acid	1:1:(1)
16	L-Pro-Ur	L-proline	Urea	1:1:(3)
17	L-Pro-Am		Acetamide	1:1:(2)
18	L-Pro-Glu		D-Glucose	1:1:(5)
19	L-Pro-Suc		Sucrose	2:1:(10)
20	L-Pro-Sor		D-Sorbitol	1:2:(4)
21	L-Pro-Gly		Glycerol	2:5
22	L-Pro-Ca		Citric Acid	1:1:(2)
23	L-Pro-Lac		Lactic Acid	1:3
24	L-Pro-Maa		DL-Malic Acid	1:1:(1)
25	L-Pro-Laa		L-Ascorbic Acid	1:1

Table S2. Extraction yields of PUE, 3-MPR and PRX from RP using NaDESs and traditional solvents (values are expressed as mean ± SD)

Entry	Solvent	PUE (mg/g)	3-MPR (mg/g)	PRX (mg/g)
**1**	water	44.9±1.7	5.7±0.3	2.9±0.5
**2**	methanol	45.5±3.9	5.8±0.7	3.8±0.7
**3**	ChCl-Ur	51.0±2.8	7.4±0.2	4.4±0.6
**4**	ChCl-Am	53.9±2.9	6.8±0.4	4.3±0.6
**5**	ChCl-Glu	57.4±0.7	6.6±0.2	4.0±0.6
**6**	ChCl-Ma	53.1±1.5	7.2±1.3	4.5±1.1
**7**	ChCl-Xyl	52.3±5.0	6.4±1.3	4.0±1.0
**8**	ChCl-Gly	54.6±8.9	6.0±1.4	5.2±0.8
**9**	ChCl-Lac	51.4±6.8	6.6±0.5	4.6±0.5
**10**	ChCl-Maa	66.2±0.4	6.4±0.4	3.6±0.2
**11**	Bet-Ur	50.4±2.8	7.8±0.3	5.0±0.7
**12**	Bet-Ma	60.7±1.0	7.2±0.5	4.4±0.7
**13**	Bet-Suc	55.9±1.9	7.4±0.6	4.5±0.7
**14**	Bet-Xyl	55.7±3.9	7.0±0.3	4.5±0.4
**15**	Bet-Gly	54.0±2.3	7.6±1.6	4.8±1.4
**16**	Bet-Lac	52.9±2.3	8.8±1.1	5.0±0.7
**17**	Bet-Maa	70.4±1.6	6.6±0.5	4.1±0.7
**18**	L-Pro-Ur	53.1±2.9	8.1±1.0	4.9±0.6
**19**	L-Pro-Am	59.1±3.9	7.3±1.0	4.2±0.3
**20**	L-Pro-Glu	56.2±1.0	6.7±0.7	3.5±0.3
**21**	L-Pro-Suc	56.9±8.5	7.7±0.3	4.4±0.5
**22**	L-Pro-Sor	55.2±1.0	8.3±1.6	5.3±0.4
**23**	L-Pro-Gly	62.4±2.1	8.3±1.3	5.1±0.4
**24**	L-Pro-Ca	67.7±3.1	9.7±0.8	5.5±0.5
**25**	L-Pro-Lac	59.5±3.0	6.9±0.6	4.5±0.9
**26**	L-Pro-Maa	74.0±2.8	6.9±0.4	4.0±0.1
**27**	L-Pro-Laa	54.0±2.5	6.8±0.4	3.8±0.1

Table S3. Extraction yields of PUE, 3-MPR and PRX from RP using L-Pro-Maa under different conditions (values are expressed as mean±SD)

Extraction Condition		PUE (mg/g)	3-MPR (mg/g)	PRX (mg/g)
A. S/L Ratio (mg/mL)	25	77.1	10.4	7.1
	50	47.0	6.6	4.5
	100	32.4	5.0	3.6
	150	26.1	5.2	3.8
	200	21.5	5.3	3.7
B. DES Content (%)	15	76.4	10.8	7.0
	30	82.7	10.5	7.2
	60	86.3	14.2	9.4
	75	77.1	10.4	7.1
C. Extraction Time (min)	15	38.3	4.9	3.4
	30	77.1	10.4	7.1
	45	75.0	10.4	6.8
D. Extraction Temperature(°C)	30	75.0	10.3	6.7
	40	93.5	16.7	11.2
	50	77.1	10.4	7.1
	60	75.9	11.6	8.4
	70	58.6	7.9	5.4

Table S4. Calibration curves, linear ranges, LOD and LOQ for analytes by HPLC

No.	Wavelength (nm)	Calibration Curve	R^2	Linear Range (mg/L)	LOD (mg/L)	LOQ (mg/L)
PUE		y = 43724x + 94419	0.9992	100-1000	3.9	12.0
3-MPR	254	y=65.556x+139.88	0.9998	5-200	1.6	4.7
PRX		y=75.047x+65.267	0.9991	1-100	0.3	1.0

* A calibration curve was obtained by injecting standard solutions of PUE at different concentrations, 1000, 750, 500, 250 and 100 mg/L. The calibration curve as follow: y = 43,724x + 94,419 (R^2=0.9992).

* A calibration curve was obtained by injecting standard solutions of 3-MPR at different concentrations, 200, 100, 50, 25 and 5 mg/L. The calibration curve as follow: y = 65.556x + 139.88 (R^2=0.9998).

* A calibration curve was obtained by injecting standard solutions of PRX at different concentrations, respectively 100, 50, 25, 10 and 1 mg/L. The calibration curve as follow: y = 75.047x+65.267 (R^2=0.9991).

* LOD and LOQ was determined following references:

Reference:

[1] Mannino G, Di Stefano V, Lauria A, et al. Vaccinium macrocarpon (cranberry)-based dietary supplements: Variation in mass uniformity, proanthocyanidin dosage and anthocyanin profile demonstrates quality control standard needed[J]. Nutrients, 2020, 12(4).

[2] Sarkar M, Khandavilli S, Panchagnula R. Development and validation of rp-hplc and ultraviolet spectrophotometric methods of analysis for the quantitative estimation of antiretroviral drugs in pharmaceutical dosage forms[J]. J Chromatogr B Analyt Technol Biomed Life Sci, 2006, 830(2): 349-354.

Figure S1. HPLC chromatogram of aqueous extract of RP (wavelength=254nm)

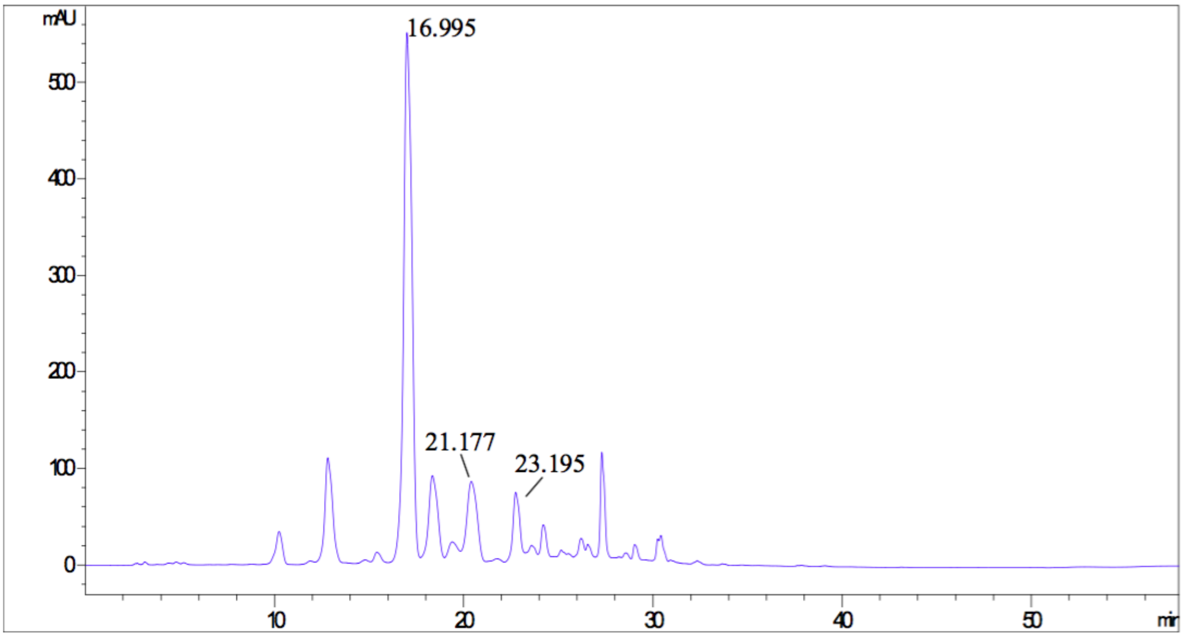

Figure S2. HPLC chromatogram of L-Pro-Maa extract of RP (wavelength=254nm)

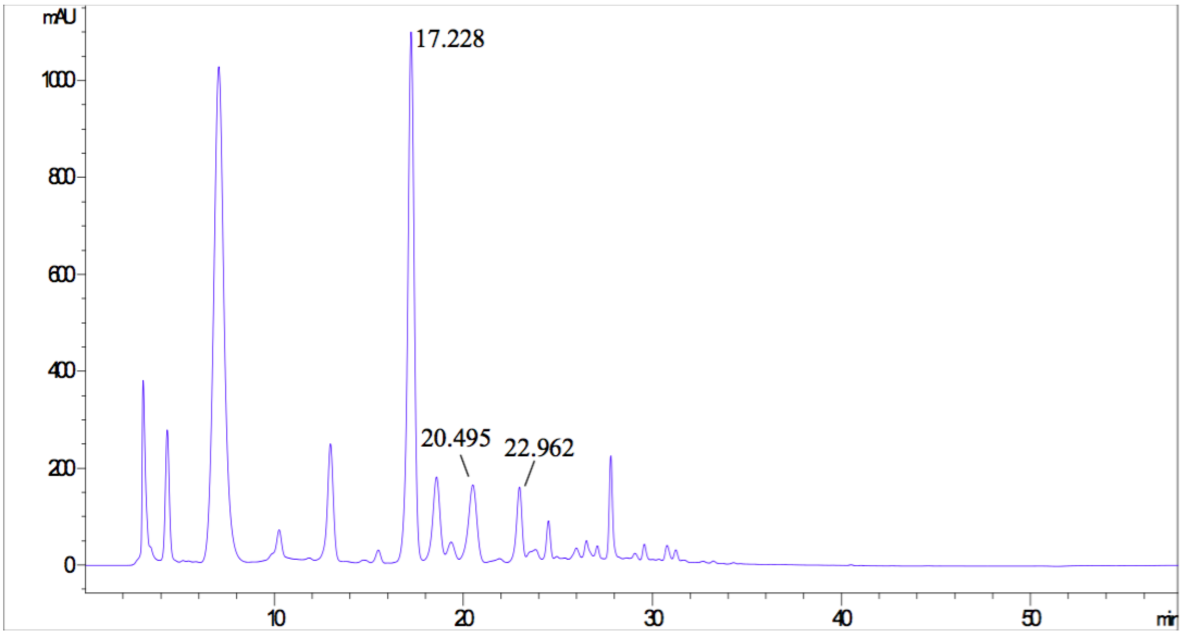

Figure S3. HPLC chromatogram of methanol extract of RP（wavelength=254nm）

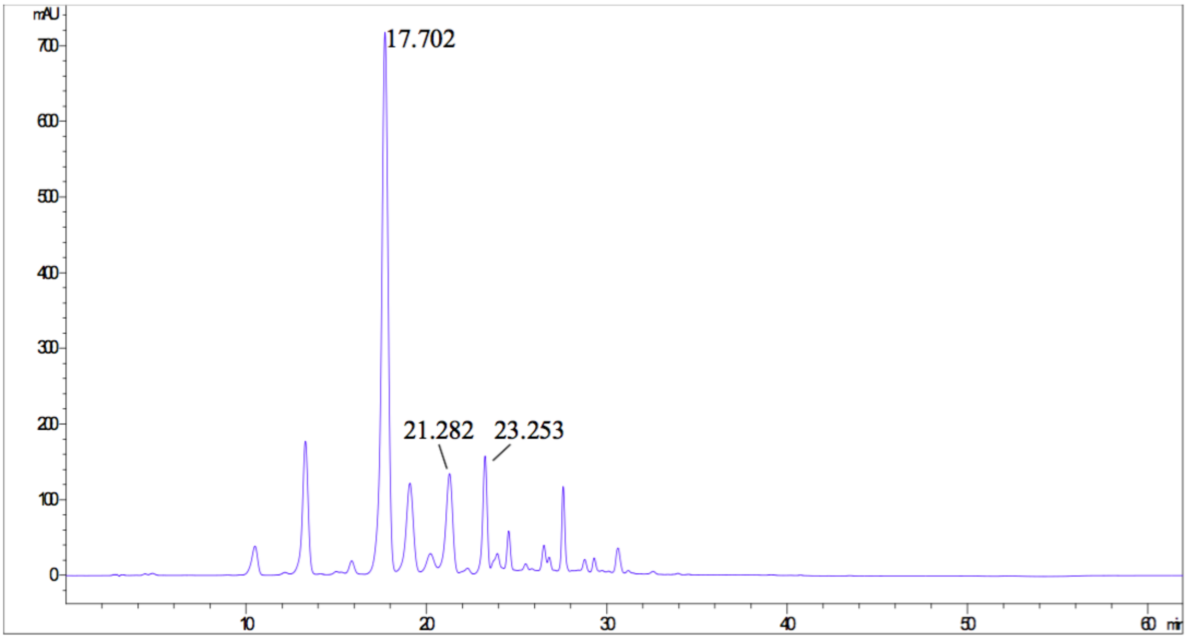

Supplement: Supplementary file 1 [file molecules-26-00729-s001.pdf]
